# Damage Detection on Sudden Stiffness Reduction Based on Discrete Wavelet Transform

**DOI:** 10.1155/2014/807620

**Published:** 2014-06-01

**Authors:** Bo Chen, Zhi-wei Chen, Gan-jun Wang, Wei-ping Xie

**Affiliations:** ^1^Key Laboratory of Roadway Bridge and Structural Engineering, Wuhan University of Technology, Wuhan 430070, China; ^2^School of Architecture and Civil Engineering, Xiamen University, Xiamen 361005, China; ^3^Zhongshan Power Supply Bureau, Guangdong 528400, China

## Abstract

The sudden stiffness reduction in a structure may cause the signal discontinuity in the acceleration responses close to the damage location at the damage time instant. To this end, the damage detection on sudden stiffness reduction of building structures has been actively investigated in this study. The signal discontinuity of the structural acceleration responses of an example building is extracted based on the discrete wavelet transform. It is proved that the variation of the first level detail coefficients of the wavelet transform at damage instant is linearly proportional to the magnitude of the stiffness reduction. A new damage index is proposed and implemented to detect the damage time instant, location, and severity of a structure due to a sudden change of structural stiffness. Numerical simulation using a five-story shear building under different types of excitation is carried out to assess the effectiveness and reliability of the proposed damage index for the building at different damage levels. The sensitivity of the damage index to the intensity and frequency range of measurement noise is also investigated. The made observations demonstrate that the proposed damage index can accurately identify the sudden damage events if the noise intensity is limited.

## 1. Introduction

The widely used vibration-based damage assessment methods require modal properties that are obtained from signals via the traditional Fourier transform (FT) [1, 2]. There are a few inherent characteristics of the FT that might affect the accuracy of damage identification. The FT is not able to present the time dependency of signals and it cannot capture the evolutionary characteristics that are commonly observed in the signals measured from naturally excited structures [3, 4]. This factor adds difficulties to the implementation aspect of the FT-based damage detection techniques. Wavelet transform (WT) can be viewed as an extension of the traditional FT with the adjustable window location and size which has recently emerged as a promising tool for structural health monitoring (SHM) and damage detection due to its inherent properties [5–7].

The earliest work on applying wavelet analysis in SHM dated back to the work of Yamamato and his group in 1995. The cumulative damage of a building with bilinear restoring force subjected to a real earthquake ground motion was estimated in terms of the accumulated ductility ratio, which is related to the number of spikes in the wavelet results [8, 9]. The wavelet approach for online detection of a sudden stiffness loss was studied and the results were compared with other approaches such as a neural network based online approximation technique and the empirical mode decomposition (EMD) method. Hou et al. [10] proposed a wavelet-based approach to identify the damage time instant and damage location of a simple structural model with breakage springs. By decomposing a vibration signal in the time domain using wavelet analysis, the discontinuity in the signal will form a signal feature, termed damage spike, in the wavelet details. Sohn et al. [11] incorporated wavelet transforms with the Holder exponent to capture the time varying nature of discontinuities. Vincent et al. [12] and Yang et al. [13, 14] used empirical mode decomposition, developed by Huang et al. [15, 16], to decompose the vibration signal to capture the signal discontinuity. Xu and Chen [17] carried out experimental studies on the applicability of EMD for detecting structural damage caused by a sudden change of structural stiffness. Chen and Xu [18] proposed two online detection approaches to the sudden damage detection.

The sudden stiffness reduction in a structure may cause the signal discontinuity in the acceleration responses close to the damage location at the damage time instant. In reality, the signal discontinuity around damage instant due to sudden stiffness loss can be taken as a kind of signal singularity and can be detected by the WT. However, the severity of damage events cannot be depicted by the current developed WT based detection approaches. To this end, the damage detection on sudden stiffness reduction of building structures has been actively investigated in this study. The signal discontinuity of the structural acceleration responses of an example building is extracted based on the discrete wavelet transform (DWT). It is proved that the variation of the first level detail coefficients of the WT at damage instant is linearly proportional to the magnitude of the stiffness reduction. A new damage index is developed and implemented in this paper to detect the damage time instant, location, and severity of a structure due to a sudden change of structural stiffness. Numerical simulation using a five-story shear building under different types of excitation is carried out to assess the effectiveness and reliability of the proposed damage index for the building at different damage levels. The sensitivity of the damage index to the intensity and frequency range of measurement noise is also investigated. The made observations demonstrate that the proposed damage index can accurately identify the damage time instant and location in the building due to a sudden loss of stiffness. The relation between the damage severity and the proposed damage index is linear. The proposed damage index can identify the damage events from the contaminated acceleration responses if the noise intensity is limited.

## 2. Wavelet Transform

Morlet and Grossmann initially proposed wavelet theory and Meyer developed the mathematical foundations of wavelets. The two America-based researchers, Daubechies [19, 20] and Mallat [21], changed this by defining the connection between wavelets and digital signal processing. Wavelets have been applied to a number of areas, including data compression, image processing, and time-frequency spectral estimation. A mother wavelet *ψ*(*t*) is a waveform that has limited duration and an average value of zero and the wavelet kernel can be expressed by

(1) $$\begin{array}{c} \psi_{a,b}\left(t\right)=\frac{1}{\sqrt{a}}\psi \left(\frac{t-b}{a}\right), \end{array}$$

where *a* and *b* are dilation and translation parameters, respectively. Both are real numbers and *a* must be positive. Similar to the short time Fourier transform, one can analyze square-integrable function *f*(*t*) with wavelet transform, which decomposes a signal in the time domain into a two-dimensional function in the time-scale plane (*a*, *b*) as follows:

(2) $$\begin{array}{c} C\left(a,b\right)=\overset{+\infty }{\underset{-\infty }{\int }}f\left(t\right)\psi_{a,b}^{}\left(t\right)dt=\frac{1}{\sqrt{a}}\overset{+\infty }{\underset{-\infty }{\int }}f\left(t\right)\psi \left(\frac{t-b}{a}\right)dt. \end{array}$$

The term frequency instead of scale has been used in order to aid in understanding, since a wavelet with large-scale parameter is related to low-frequency content component and vice versa. The mother wavelet *ψ*(*t*) should satisfy the following admissibility condition to ensure existence of the inverse wavelet transform such as

(3) $$\begin{array}{c} C_{\psi }=\overset{+\infty }{\underset{-\infty }{\int }}\frac{{\left|\hat{\psi }\left(\omega \right)\right|}^{2}}{\left|\omega \right|}d\omega <+\infty , \end{array}$$

where $\hat{\psi }(\omega )$ is the Fourier transform of *ψ*(*t*). The existence of the integral in (3) requires that

(4) $$\begin{array}{c} \hat{\psi }\left(0\right)=0,\;\text{i}.\text{e}.,\;\overset{+\infty }{\underset{-\infty }{\int }}\psi \left(x\right)dx=0. \end{array}$$

The signal *f*(*t*) can be reconstructed by an inverse wavelet transform of *C*(*a*, *b*) as defined by

(5) $$\begin{array}{c} f\left(t\right)=\frac{1}{C_{\psi }}\overset{+\infty }{\underset{a=-\infty }{\int }}\overset{+\infty }{\underset{b=-\infty }{\int }}C\left(a,b\right)\psi_{a,b}^{}\left(\frac{t-b}{a}\right)\frac{1}{a^{2}}da\;db. \end{array}$$

The calculating wavelet coefficients at every possible scale will generate a lot of redundant data. A discrete version of the wavelet is often utilized by discretizing the dilation parameter *a* and the translation parameter *b* in real signal processing. The procedure becomes much more efficient if dyadic values of *a* and *b* are used. That is,

(6) $$\begin{array}{c} a=2^{j};\;b=2^{j}k\;j,k\in Z, \end{array}$$

where *Z* is a set of integers. This sampling of the coordinates (*a*, *b*) is referred to as dyadic sampling because consecutive values of the discrete scales differ by a factor of 2. Using the discrete scales of WT, one can define the discrete wavelet transform (DWT) [21] as follows:

(7) $$\begin{array}{c} C_{j,k}^{}=\overset{+\infty }{\underset{-\infty }{\int }}f\left(t\right)\psi_{j,k}^{}\left(t\right)dt=2^{-j/2}\overset{+\infty }{\underset{-\infty }{\int }}f\left(t\right)\psi \left(2^{-j}t-k\right)dt. \end{array}$$

The signal resolution is defined as the inverse of the scale 1/*a* = 2^−*j*^, and the integer *j* is referred to as the level. The signal can be reconstructed from the wavelet coefficients *C*_*j*,*k*_ and the reconstruction algorithm is called the inverse discrete wavelet transform as follows:

(8) $$\begin{array}{c} f\left(t\right)=\overset{+\infty }{\underset{j=-\infty }{\sum }}\;\overset{+\infty }{\underset{k=-\infty }{\sum }}C_{j,k}^{}2^{-j/2}\psi \left(2^{-j}t-k\right). \end{array}$$

Another function *ϕ*(*t*), referred to as the scaling function, is important for the numerical implementation of the fast wavelet transform [19]. Suppose now that the dyadic scale is used for *a* and *b*, and consider a reference level *J*. Applying (7) for this case one obtains a set of coefficients as follows:

(9) $$\begin{array}{c} cD_{J}^{}\left(k\right)=\overset{+\infty }{\underset{-\infty }{\int }}f\left(t\right)\psi_{J,k}^{}\left(t\right)dt. \end{array}$$

The coefficient *cD*_*j*_(*k*) is known as the level-*J* detail coefficients. Using the dyadic scale level *J* yields the level-*J* approximation coefficients as follows:

(10) $$\begin{array}{c} cA_{J}^{}\left(k\right)=\overset{+\infty }{\underset{-\infty }{\int }}f\left(t\right)\phi_{J,k}^{}\left(t\right)dt. \end{array}$$

In the DWT, a signal can be represented by its approximations and details. The detail at level *j* is defined as

(11) $$\begin{array}{c} D_{j}\left(t\right)=\overset{+\infty }{\underset{k=-\infty }{\sum }}cD_{j}^{}\left(k\right)\psi_{j,k}^{}\left(t\right) \end{array}$$

and the approximation at level *j* is defined as

(12) $$\begin{array}{c} A_{J}\left(t\right)=\overset{+\infty }{\underset{k=-\infty }{\sum }}cA_{J}^{}\left(k\right)\phi_{j,k}^{}\left(t\right). \end{array}$$

It becomes obvious that

(13) $$\begin{array}{c} A_{J-1}=A_{J}+D_{J},\\ f\left(t\right)=A_{J}\left(t\right)+\underset{j\leq J}{\sum }D_{j}\left(t\right). \end{array}$$

## 3. Signal Feature due to Sudden Damage

**Figure 1:**
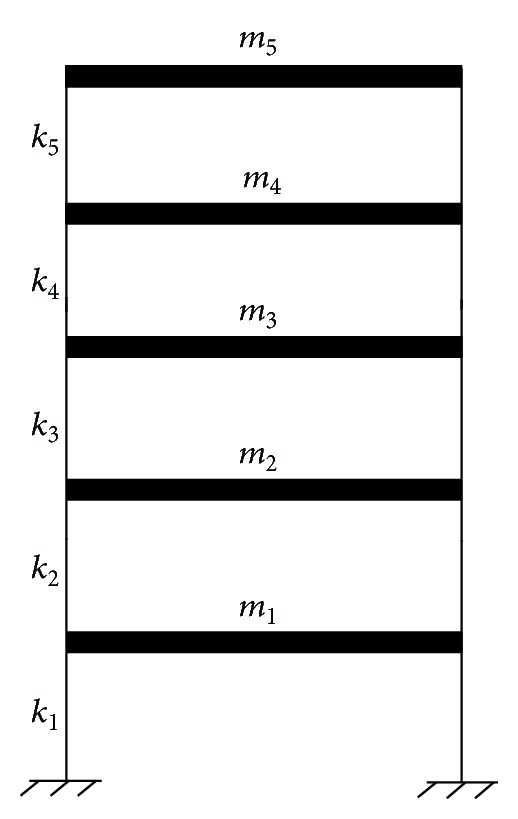
Elevation of a five-story building model.

The dynamic responses of a five-story shear building subjected to a sudden stiffness reduction at its first story under three different external excitations are computed. The mass and horizontal stiffness of the undamaged building are uniform for all stories as shown in Figure 1. The mass and horizontal stiffness of the each floor are *m* = 1.3 × 10^6^ kg and *k* = 4.0 × 10^9^ N/m, respectively. The Rayleigh damping assumption is adopted to construct the structural damping matrix, and the damping ratios in the first two modes of vibration of the building are set as 0.05. The original building is supposed to suffer a sudden 20% stiffness reduction in the first story with the horizontal stiffness reducing from 4.0 × 10^9^ N/m to 3.2 × 10^9^ N/m, while the horizontal stiffness in other stories remains unchanged. The frequency reduction due to 20% stiffness reduction in the first story is small with a maximum reduction of no more than 5% in the first natural frequency.

The sinusoidal excitation, seismic excitation, and impulse excitation are, respectively, utilized to calculate the acceleration responses of the example building to examine the signal features due to sudden stiffness reduction. The seismic excitation used is the first 10-second portion of the El-Centro 1940 earthquake ground acceleration (S-N component) with a peak amplitude of 1.0 m/s^2^. A sinusoidal excitation expressed by the following equation with 10-second duration is assumed to act on each floor of the building:

(14) $$\begin{array}{c} f\left(t\right)=1300\cdot \sin\left(4\pi t\right)\;\left(0\leq t\leq 10\;\text{s}\right)\;\left(\text{kN}\right). \end{array}$$

An impulse excitation represented by 0.1 m/s initial velocity is supposed to occur at the first floor of the building. The damage time instant of the building is set as 6.0 s for seismic excitation and sinusoidal excitation and as 0.2 s for impulse excitation. The equation of motion of the example building with a 20% sudden stiffness reduction at its first story at the given time instant is established. The dynamic responses under each type of external excitation are computed by using the Newmark-*β* method with a time interval of 0.002 s. The two factors in the Newmark-*β* method are selected as *α* = 1/2 and *β* = 1/4 [22, 23].

**Figure 2:**
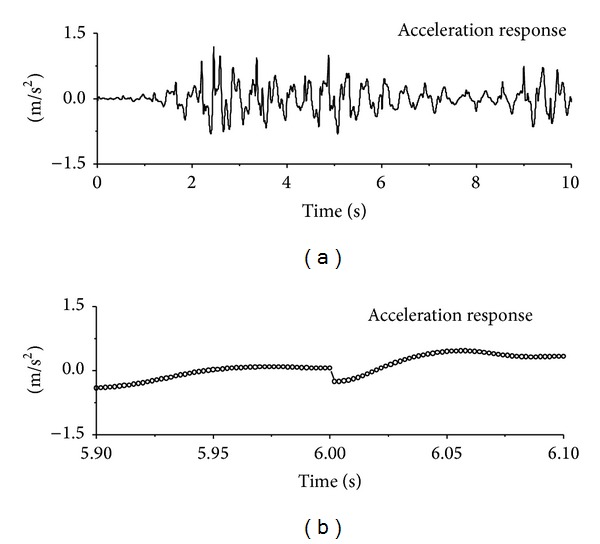
Signal discontinuity due to sudden damage (seismic excitation).

**Figure 3:**
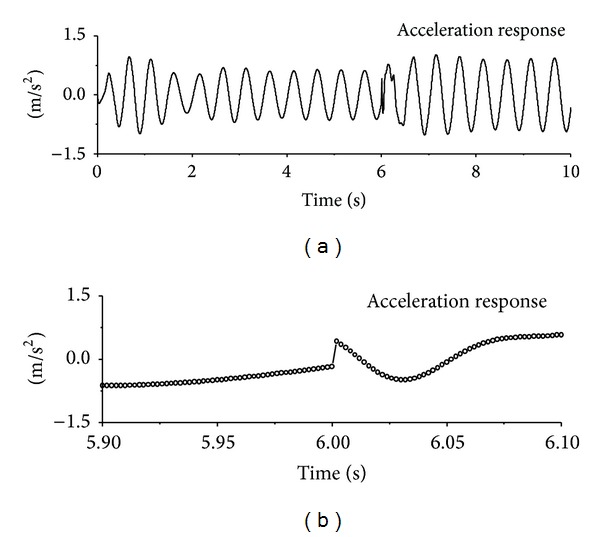
Signal discontinuity due to sudden damage (sinusoidal excitation).

**Figure 4:**
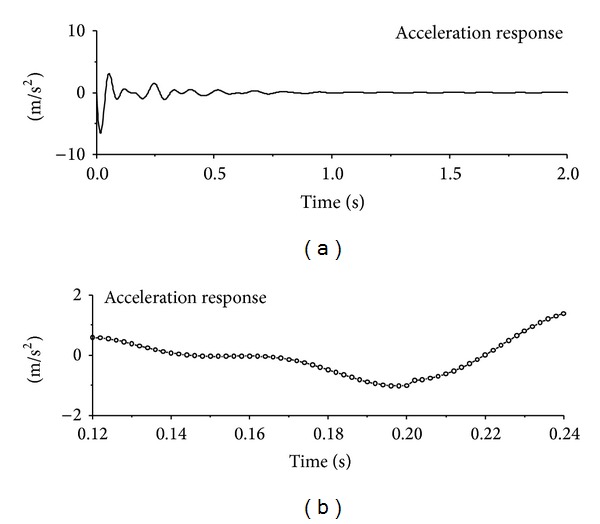
Signal discontinuity due to sudden damage (impulse excitation).

The computed acceleration time histories of the first floor under seismic excitation are displayed in Figure 2. It is difficult to find the signal feature due to sudden damage by direct visual inspection of the original acceleration responses. The 0.2-second portion of the acceleration responses is expanded to permit a close look at the signal feature due to sudden damage event. It is seen that there exists a sudden jump in the original signal at the damage time instant. The structural acceleration time histories of the first floor under sinusoidal and impulse excitation are also displayed in Figures 3 and 4, respectively. Similar to the observations made from seismic excitation, the direct inspection on original signals cannot directly find the signal feature due to sudden damage event. A detained investigation on small time portions indicates the sudden jump of original acceleration responses at damage time instants as shown in the figures. The sudden reduction of horizontal stiffness of the first floor causes a clear signal discontinuity in the acceleration response time history at the damage time instant.

**Figure 5:**
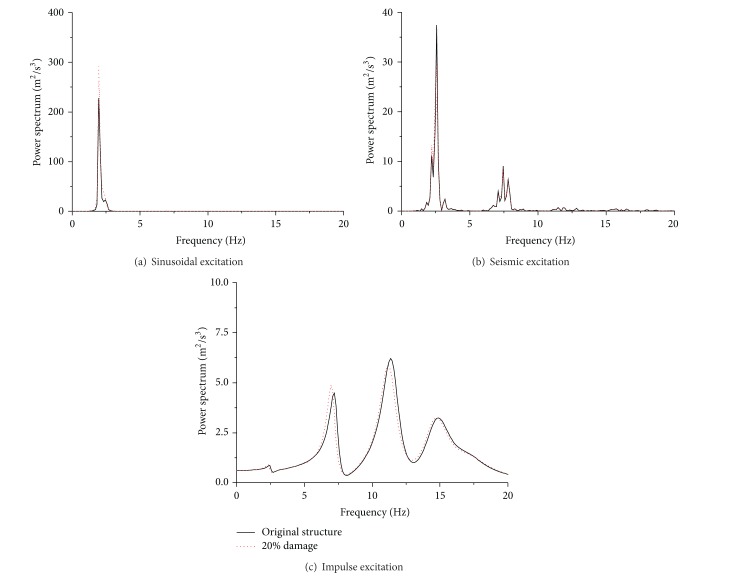
Power spectrum of acceleration responses of the first floor before and after sudden damage event.

Figure 5 displays the power spectrum of acceleration responses with and without sudden damage events. It is clear that the change in the spectrum amplitude induced by the sudden damage is very small which cannot provide the enough information to capture the damage event. Moreover, the exact damage instant still cannot be determined in the frequency domain based on the fast Fourier transform. Further inspection of the spectrum curves indicates that the structural acceleration responses present quite different spectrum properties under different external excitations. If the building is subjected to El-Centro earthquake, the power spectrum has a relatively wide frequency range, and the first two natural frequencies can be effectively identified. The impulse excitation signal, however, holds very short time interval and a very wide frequency range. The acceleration responses of the impulse excited building present very abundant frequency components and four natural frequencies can be identified from the power spectrum. To compare the spectrum components of sinusoidal, seismic, and impulse excitation, one can conclude that the structural responses subjected to impulse excitation have the most abundant high frequency components.

**Figure 6:**
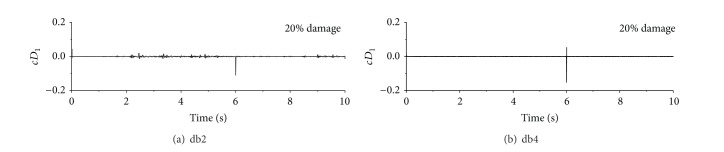
Level-1 detail coefficients of wavelet transform for acceleration responses under seismic excitation.

Since the signal discontinuity is of very high frequency, the wavelet transform is applied to decompose the original acceleration responses.  Figure 6 displays the first level detail coefficients of wavelet transform for acceleration responses under seismic excitation. It can be seen that the signal discontinuity is reserved in the first level detail coefficient only instead of in the approximation components. This is because the first level detail component often contains the highest frequency component of the original signal. To extract inherent signal feature due to sudden damage from the signal discontinuity in the original acceleration response time history, the acceleration responses of the building under each type of excitation are computed for a sudden reduction of stiffness at the first story with different damage levels and damage time instants. Similar observations can be made from the decomposed detail coefficients of the wavelet transform of the acceleration responses under sinusoidal and impulse excitations.

## 4. Damage Index

Let us consider a SDOF system subjected to a sudden stiffness reduction under impulse excitation. The mass of the system is denoted as *m*, the damping ratio *ξ* of the system is supposed to remain unchanged before and after sudden damage, and the stiffness is denoted as *k* which will have a sudden reduction at time instant *t*_*i*_ as follows:

(15) $$\begin{array}{c} k=\{\begin{array}{cc} k_{u} & \left(0\leq t\leq t_{i}\right)\\ k_{d} & \left(t_{i}<t\right), \end{array} \end{array}$$

in which *k*_*u*_ and *k*_*d*_ are the stiffness of undamaged and damaged system, respectively. The initial velocity and displacement due to the impulse excitation are assumed to be 0 and *v*_0_, respectively. The circular frequency of the system before and after sudden damage can be expressed as

(16) $$\begin{array}{c} \omega_{u}=\sqrt{\frac{k_{u}}{m}};\;\;\omega_{d}=\sqrt{\frac{k_{d}}{m}}. \end{array}$$

Define a frequency reduction coefficient *α* that varies from 0 to 1 as follows:

(17) $$\begin{array}{c} \omega_{d}=\alpha \cdot \omega_{u}\;\left(0<\alpha <1\right). \end{array}$$

The stiffness reduction can be expressed as

(18) $$\begin{array}{c} \Delta k=k_{d}-k_{u}=m\left(\omega_{d}^{2}-\omega_{u}^{2}\right)=m\omega_{u}^{2}\left(\alpha^{2}-1\right). \end{array}$$

The equation of motion of the SDOF system before sudden damage is

(19) $$\begin{array}{c} \ddot{y}+2\xi \omega_{u}\ddot{y}+\omega_{u}^{2}y=0. \end{array}$$

The above equation can be solved in terms of the given initial conditions, and the structural dynamic responses are

(20) y(t)=Au(t)v0e−ξωut·1ωu1−ξ2,y˙(t)=v0e−ξωut(Bu(t)−Au(t)ξ1−ξ2),y¨(t)=−v0ωue−ξωut·[Au(t)(1−2ξ2)+2ξBu(t)1−ξ2]1−ξ2,

in which

(21) $$\begin{array}{c} A_{u}\left(t\right)=\sin\left(\omega_{u}t\sqrt{1-\xi^{2}}\right),\\ B_{u}\left(t\right)=\cos\left(\omega_{u}t\sqrt{1-\xi^{2}}\right). \end{array}$$

Let us take the time instant *t*_*i*_ as the starting point of the SDOF system after sudden damage and use a new time axis *t*_1_ = *t* − *t*_*i*_. Then, the equation of motion of the system after damage becomes

(22) $$\begin{array}{c} {\ddot{y}}_{d}+2\xi \omega_{d}{\ddot{y}}_{d}+\omega_{d}^{2}y_{d}=0\;\left(t>t_{i}\right). \end{array}$$

The initial conditions for (10) can be expressed as

(23) yd(0)=y(ti)=Au(ti)v0e−ξωuti·1ωu1−ξ2,y˙d(0)=y˙(ti)=v0e−ξωuti(Bu(ti)−Au(ti)ξ1−ξ2).

The damping ratio of a civil engineering structure is often very small; that is, $\sqrt{1-\xi^{2}}\approx 1$. The acceleration response at the time instant *t*_1_ is

(24) y¨d(t1) =v0ωde−ξ(ωdt1+ωuti)(ξ2−1)ωu×{ωdAu(ti)[Bd(t1)1−ξ2−Ad(t1)ξ]+ωuAd(t1)(2ξ2−1)(Au(ti)ξ−Bu(ti)1−ξ2)−2ωuξBd(t1)[Au(ti)ξ1−ξ2+Bu(ti)(ξ2−1)]}.

Furthermore, the time interval should be very small to describe the sudden stiffness reduction properly; thus

(25) $$\begin{array}{c} \Delta t=t_{i+1}-t_{i}⟶0,\\ A_{d}\left(\Delta t\right)=\sin\left(\omega_{d}\Delta t\sqrt{1-\xi^{2}}\right)\approx 0,\\ B_{d}\left(\Delta t\right)=\cos\left(\omega_{d}\Delta t\sqrt{1-\xi^{2}}\right)\approx 1. \end{array}$$

Therefore, the acceleration response at the time instant *t*_*i*+1_ is

(26) y¨(ti+1)=y¨d(Δt)=v0ωde−ξωuti(ξ2−1)ωu ×{ωdAu(ti)1−ξ2−2ξωu×[Au(ti)ξ1−ξ2+Bu(ti)(ξ2−1)]}.

The first level detail coefficients of wavelet transform of the acceleration responses before sudden damage $cD_{1}^{\ddot{y}(t)}$ can be expressed as

(27) $$\begin{array}{c} cD_{1}^{\ddot{y}(t)}\left(k\right)=\overset{+\infty }{\underset{-\infty }{\int }}\ddot{y}\left(t\right)\psi_{1,k}^{}\left(t\right)dt. \end{array}$$

The first level detail coefficients of wavelet transform of the acceleration responses after sudden damage $cD_{1}^{{\ddot{y}}_{d}(t_{1})}$ can be expressed as

(28) $$\begin{array}{c} cD_{1}^{{\ddot{y}}_{d}(t_{1})}\left(k\right)=\overset{+\infty }{\underset{-\infty }{\int }}{\ddot{y}}_{d}\left(t_{1}\right)\psi_{1,k}^{}\left(t_{1}\right)dt_{1}. \end{array}$$

The variation of first level detail coefficients of the WT before and after the sudden damage event can be given as

(29) $$\begin{array}{c} cD_{1}^{i+1}\left(k\right)-cD_{1}^{i}\left(k\right)=\overset{+\infty }{\underset{-\infty }{\int }}\left(\ddot{y}\left(t_{i+1}\right)-\ddot{y}\left(t_{i}\right)\right)\psi_{1,k}^{}\left(t\right)dt. \end{array}$$

Considering that the damping ratio of a civil engineering structure is often very small, the above expression can be simplified as

(30) cD1i+1(k)−cD1i(k) =−Δkv0mωu∫−∞+∞e−ξωuti·sin(ωuti1−ξ2)ψ1,k(t)dt.

The above equation reveals that the variation of first level detail coefficients of the WT before and after a sudden damage event is approximately linear to the sudden stiffness reduction for given initial velocity, damage instant, and structural parameters before damage. If the time interval Δ*t* for sudden damage is further regarded as a fixed value, (30) indicates that the acceleration response discontinuity due to sudden stiffness reduction can be reflected by the variation rate of first level detail coefficients of the wavelet transform at damage instant. A damage index, DI_*i*_, is defined to reflect the signal discontinuity due to sudden damage at the time instant *t*_*i*_ as follows:

(31) $$\begin{array}{c} \text{D}{\text{I}}_{i}=\left|\frac{cD_{1}^{i+1}\left(k\right)-cD_{1}^{i}\left(k\right)}{\Delta t}\right|\;\left(k=2,3,\ldots ,n-1\right), \end{array}$$

where Δ*t* = *t*_*k*+1_ − *t*_*k*_ and *n* is the total number of time intervals for the whole response time history. This damage index is computed in the time domain and it is an instantaneous index suitable for online structural health monitoring application. The linear relationship between the proposed damage index and the sudden stiffness reduction can be observed as follows:

(32) $$\begin{array}{c} \text{D}{\text{I}}_{i}\propto \left|\Delta k\right|. \end{array}$$

## 5. Damage Detection

### 5.1. Selection of Mother Wavelet

To examine the feasibility of the proposed damage index and damage detection approaches, the first floor of the five-story building is supposed to suffer different levels of the sudden stiffness reduction, but the sudden damage occurs at the same time. Six damage scenarios are considered in the numerical investigation. Listed in Table 1 are the damage severities and the five natural frequencies of the building before and after the sudden damage. It is seen from Table 1 that the stiffness reduction in the first story of the building affects mainly lower natural frequencies. It is noted that if the stiffness reduction in the first floor is less than 10%, the maximum frequency change is no more than 2%. In addition, the variations of the higher mode shapes are much smaller than those of the lower mode shapes.

**Figure 7:**
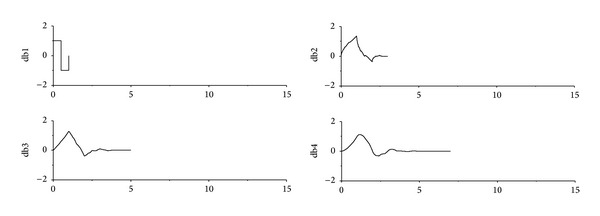
The Daubechies wavelets (db1–db4).

Wavelet transform can be utilized to detect the signal singularity due to sudden stiffness change. While the detection efficiency depends on many factors such as wavelet vanishing moments, supporting length in the time domain, frequency components of original acceleration responses, and signal noise. Thus, three different Daubechies mother wavelets db1, db2, and db4 are utilized to examine the effects of properties of mother wavelets on the detection on the structural sudden damage. The vanishing moments of the db1, db2, and db4 wavelets are 1, 2, and 4, respectively, and they have the gradually increased supporting length as plotted in Figure 7. The basic principles of wavelet transform prove that the longer the wavelet supporting length is, the finer the distinguishing ability in the frequency domain is. Therefore, the mother wavelet with long supporting length is more suitable for detecting the higher frequency components in the original signal.

To examine the feasibility of the proposed damage index and damage detection approaches for identifying damage events, the acceleration responses of the aforementioned five-story shear building to the seismic excitation, sinusoidal excitation, and impulse excitation are computed, respectively. The building is subject to a 20% sudden stiffness reduction at times 6.0 s, 6.0 s, and 0.2 s in the first story of the building under seismic excitation, sinusoidal excitation, and impulse excitation, respectively. The time step used in the computation is 0.002 seconds.

**Figure 8:**
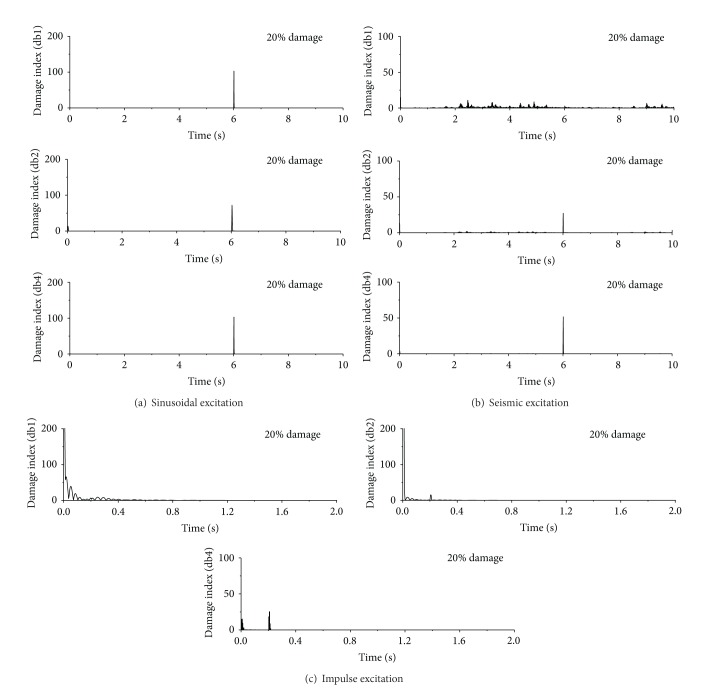
Damage detection using different db wavelets.

Shown in Figure 8 are the damage detection results using db1, db2, and db4 for 20% sudden stiffness reduction, respectively. It can be seen from Figure 8(a) that, no matter which mother wavelet is used, the damage index of the first floor is very large only at time *t* = 6.0 seconds, which is exactly the moment when the stiffness of the first story is suddenly reduced by 20%. The damage indices of the first floor at all other time instants are very small so that the damage index at time *t* = 6.0 seconds looks like a spike. Therefore, the damage time instant can be easily identified by the occurrence time of the sharp damage index. It is demonstrated that the DWT based approach using all the three Daubechies wavelets can accurately detect the damage time instant of the building subjected to sinusoidal excitation. For the building excited by El-Centro ground motion, DWT using db1 wavelet fail to detect damage instant while the approach using db2 and db4 wavelet successfully captures the damage events. For the impulse excited case, only the DWT using db4 wavelet can accurately detect the damage instant due to sudden stiffness change. In reality, the sudden stiffness loss will cause a sudden jump in acceleration responses at damage instant which may commonly introduce high frequency components into the original response signals. The crucial procedure in detecting sudden damage is to extract the high frequency components from original acceleration responses using the wavelet transform. The frequency components of acceleration responses of building subjected to sinusoidal excitation are quite simple and the high frequency signal induced by sudden damage is quite different from other signal components. All the three selected wavelets can easily detect the signal singularity and damage event. As far as the seismic excited damage building is concerned, the acceleration responses contain more high frequency components than those induced by sinusoidal excitations. The distinguishing ability in the frequency domain of the db1 wavelet is coarse due to its short supporting length in the time domain, which makes it impossible to capture the sudden damage event under seismic excitations.

The damage events can be captured by using the db2 and db4 wavelets due to their finer distinguishing ability than db1 wavelet, in particular in high frequency range. The damage events of the example building under impulse excitations are more difficult to be detected because abundant high frequency components of acceleration responses may overlap the high frequency signal induced by sudden stiffness reduction. If the extent of the damage event is minor, the energy of damage signal is too small to be reflected to the decomposed wavelet coefficients. The comparison among different mother wavelets indicates that only the db4 wavelet with fine frequency distinguishing ability can accurately capture the damage event of the building under impulse excitation.

### 5.2. Damage Time Instant

**Figure 9:**
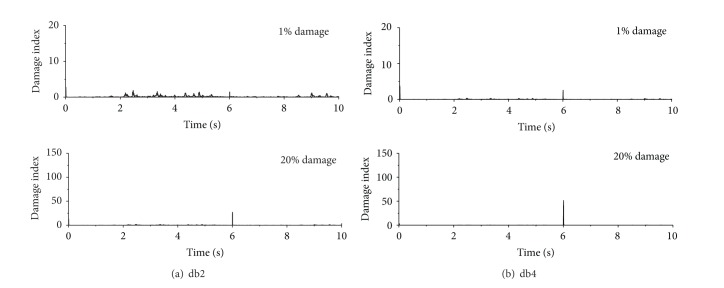
Damage detection using different db wavelets under seismic excitation.

The first floor of the five-story building is supposed to suffer different levels of sudden stiffness reduction, but the sudden reduction occurs at the same time. Two mother wavelets, namely, db2 and db4, are utilized in this section to study their performance for different damage extents as shown in Figure 9. It is clear that the db2 wavelet can accurately capture the damage features without noise contamination. For small damage cases (1% damage), the energy of damage signal is very small and the detail coefficients of the damage signal are too small to form a distinct spike at damage instant. The db4 wavelet with stronger frequency distinguishing ability can detect the minor damage event. Therefore, the damage detection on sudden stiffness reduction is carried out based on db4 wavelet in the following sections.

**Figure 10:**
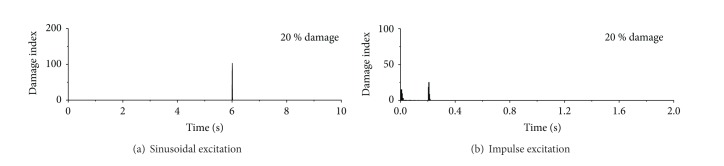
Damage detection using db4 wavelets.

The variations of damage index with time under sinusoidal excitation and impulse excitation using db4 wavelet are displayed in Figure 10. The building is subject to the same damage severity at the first story only, but it occurs at time *t* = 6.0 seconds for sinusoidal excitation and at time *t* = 0.2 seconds for impulse excitation. Again, the sharp damage index appears only at the moment of sudden stiffness reduction at the first floor. Thus, the damage time instant can be easily captured from the observed occurrence time of the sharp damage index. While the detection effects reduce with the decreasing damage extent. This is because the signal energy of the minor damage extent holds little information about damage event and the projected wavelet coefficients are too small to form a distinct spike at damage instant. Under this circumstance, the mother wavelet with higher vanishing moments and longer supporting length also cannot improve the damage detection efficiency.

### 5.3. Damage Location

**Figure 11:**
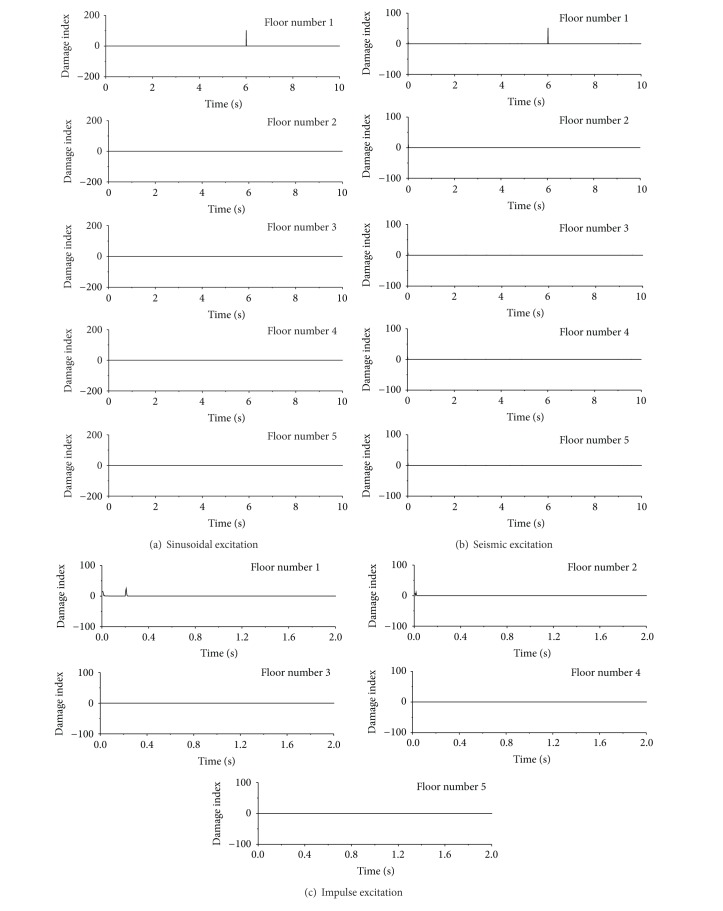
Damage detection for each floor using db4 wavelet.

Figure 11 shows the variations of damage index with time for each floor of the building under the sinusoidal, seismic, and impulse excitations. It is seen from Figure 11(a) that the damage index of the first floor is very large only at time *t* = 6.0 seconds, which is exactly the moment when the stiffness of the first story is suddenly reduced by 20%. It is essential to compare the variation of wavelet coefficients based damage index of the first floor with those of the second, third, fourth, and fifth floors of the building. The sharp spike appears clearly only at the first floor, and no sharp spike emerges in other floors. Therefore, by analyzing the distribution of spike along the height of the building, the damage location can be easily identified at the first story of the building.

The variations of damage indices with time for each floor of the building are shown in Figure 11(b) for seismic excitation using the DWT. The sharp damage index appears only at the moment of sudden stiffness reduction at the first floor. Thus, the damage location can be easily captured from the observed sharp spikes and its distribution along the height of the building. Similar results are also obtained from the building subject to sinusoidal excitation. For the impulse excited case, however, the DWT based detection approach may not give satisfactory results for the building with small damage event (1% sudden stiffness reduction). This is because the signal fluctuates significantly immediately after the initial velocity and the energy of damage signal is quite weak.

### 5.4. Damage Severity

**Figure 12:**
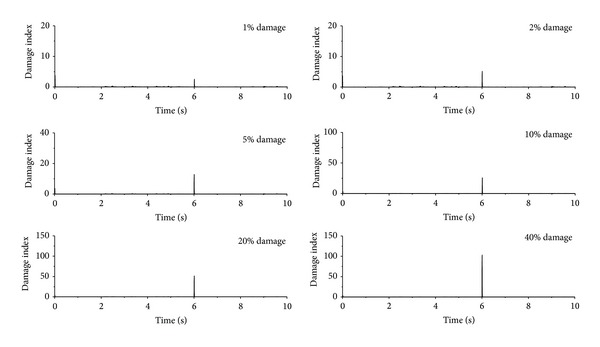
Damage detection using different db wavelets under seismic excitation.

**Figure 13:**
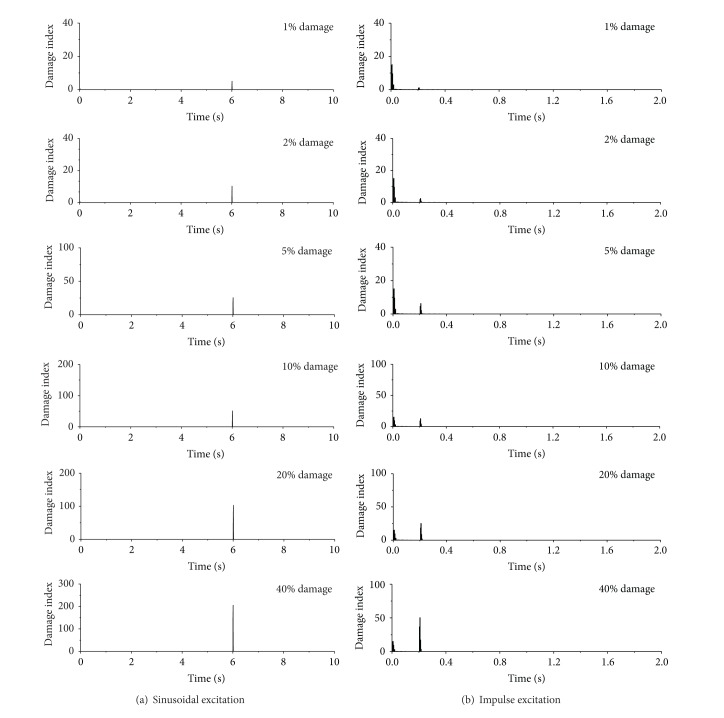
Damage detection using db4 wavelets.

The parameter investigation is carried out in this section to find the sensitivity of damage index to damage severity so as to examine the validity of the proposed damage index and damage detection approaches. The first floor of the example building is supposed to suffer different levels of sudden stiffness reduction, but the damage time instants remain unchanged. The damage indices of the first floor of the building subjected to the seismic excitation are plotted in Figure 12 for the sudden stiffness reduction from 1% to 40%. It can be seen that, even for small damage events such as 1% to 5% sudden stiffness reduction, the proposed approach can easily capture the damage features without considering noise contamination. The magnitude of the sharp damage index also increases with increasing damage severity. Similar observations can be made from the building subject to sinusoidal excitation as shown in Figure 13(a). For the building under impulse excitation, however, the proposed approaches may not provide satisfactory detection effects for the building with very small damage event (1% damage severity) as shown in Figure 13(b).

**Figure 14:**
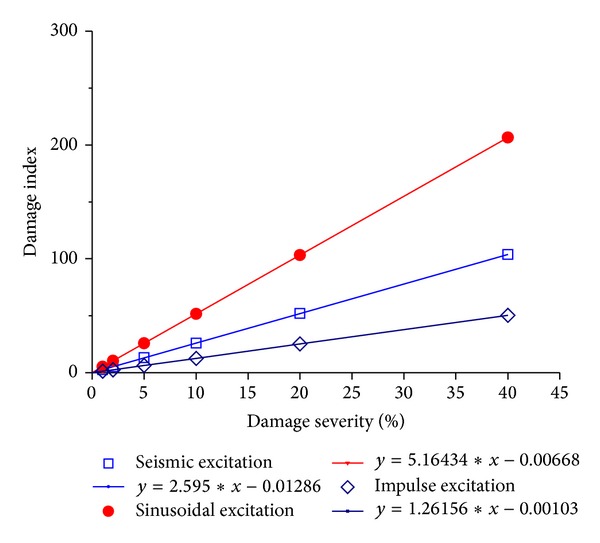
Relationship between damage index and damage severity.

The magnitudes of damage index of the example building subjected to different damage severities in the first story under seismic, sinusoidal, and impulse excitations are computed and listed in Table 2. The relationship between damage index and damage severity is also displayed in Figure 14 together with a linear fit in which *x* represents damage severity (stiffness reduction) and *y* represents damage index. It is observed that there exists a linear relationship between damage index and damage severity for the building under either seismic or sinusoidal or impulse excitation. The magnitudes of the damage index increase with the increasing extents of the stiffness reduction for a given external excitation and the slope of the linear fit is different for the building under different excitations. The proposed damage index and detection approach can be used to find the damage time instant and damage location from the measured structural responses. Then, one can measure the external excitation and input to the structural model with a sudden stiffness reduction at the identified damage location and at the identified damage time instant to determine the slope of the linear relationship between the damage severity and damage index. The linear relationship can be used finally to determine the damage severity in the actual structure in terms of the damage index identified from the actual structure.

### 5.5. Effects of Signal Noise

The effect of measurement noise on the quality of the damage detection is a key issue needed to be addressed in real application. Hou et al. [10] reported that the damage spike identified from the wavelet transform coefficients could be weakened by measurement noise and strong measurement noise could lead to the failure of damage detection. In reality, the sudden damage event may introduce a high frequency component to acceleration responses of a structure and the effects of both measurement noise intensity and frequency range on the damage detection are thereby examined [16]. The measurement noise in structural response is assumed to be a random white noise. Three frequency ranges are considered: (1) white noise with frequency range from 0 to 50 Hz; (2) white noise with frequency range from 0 to 100 Hz; and (3) white noise with frequency range from 0 to 250 Hz. The measurement noise intensity is defined as

(33) $$\begin{array}{c} \text{Noise}\;\text{intensity}=\frac{\text{RMS}\;\left(\text{noise}\right)}{\text{RMS}\;\left(\text{signal}\right)}\times 100\%. \end{array}$$

**Figure 15:**
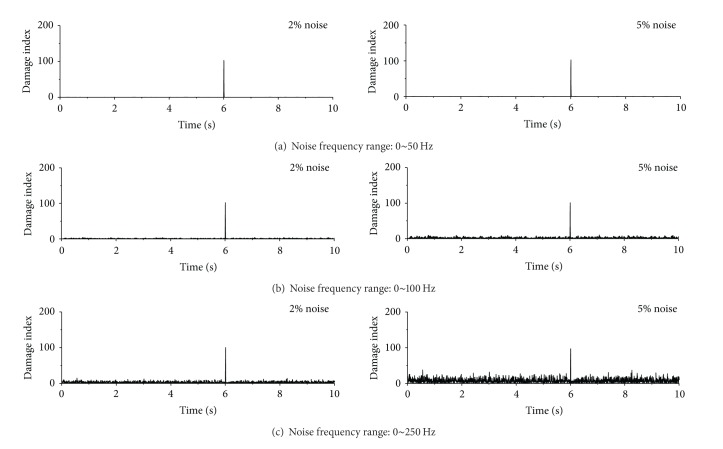
Detection results from contaminated acceleration responses (sinusoidal excitation).

**Figure 16:**
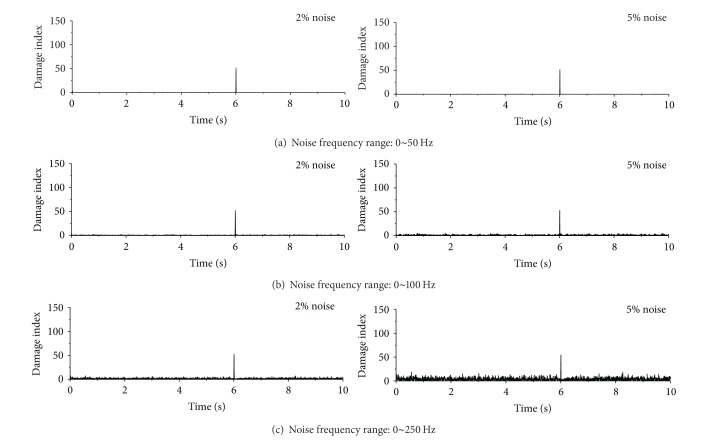
Detection results from contaminated acceleration responses (seismic excitation).

**Figure 17:**
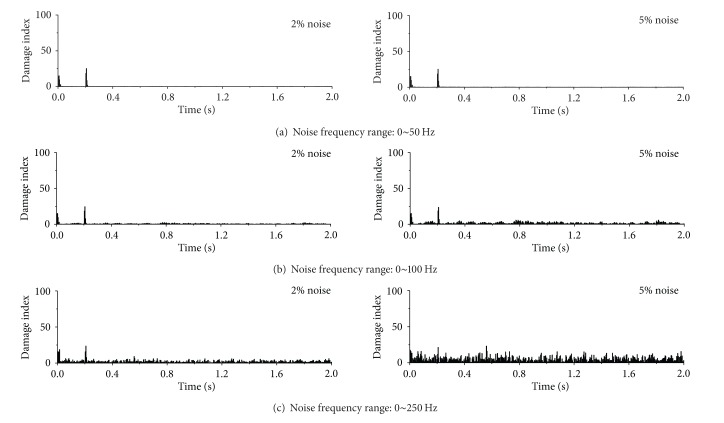
Detection results from contaminated acceleration responses (impulse excitation).

Displayed in Figures 15, 16, and 17 are damage detection results using the contaminated acceleration responses at the first floor under the sinusoidal, seismic, and impulse excitations, respectively. The noises are introduced with two noise intensities and three noise frequency ranges described as above. The sudden stiffness reduction in the first story of the building is 20%. To check the original acceleration time histories contaminated with noise, one can find that, with increasing noise frequency range, the acceleration responses are more fluctuating and the signal discontinuity at the damage time instant becomes weak. It is clear from Figure 15 that the proposed index can still identify the damage time instant from the polluted acceleration responses at the first floor for the designated two noise intensities and three noise frequency ranges when subjected to sinusoidal excitation. Furthermore, the spatial distribution of damage indices along the height of the building can indicate the damage location from the acceleration responses with noise contamination. Similar observations can be made from the detection observations of the example building subjected to the seismic excitation, as shown in Figure 16. In the impulse excitation case, however, the proposed approach fails to identify the damage time instant and damage location when the noise frequency range is from 0 to 250 Hz and the noise intensity is 5%, as shown in Figure 17.

The effects of measurement noise on the damage detection are assessed and the results indicate that both the noise intensity and the frequency range have a remarkable influence on the results from DWT based detection approach. If the noise frequency range is too narrow to overlap the sudden damage signal with high frequency components, the influence of noise intensity on detection accuracy is small. Otherwise, the detection accuracy will decrease with the increase of noise intensities.

## 6. Concluding Remarks

The damage detection on sudden stiffness reduction of building structures has been actively investigated in this study. The signal jump of the structural acceleration responses of an example building due to sudden damage is examined and the signal discontinuity is extracted based on the discrete wavelet transform. It is proved that the variation of the first level detail coefficients of the wavelet transform at damage instant is proportional to the magnitude of the stiffness reduction. In this regard, a new damage index based on the DWT is proposed in terms of the slope of the decomposed detail coefficients of wavelet transform to detect the damage time instant, damage location, and damage severity.

Extensive numerical simulations have been executed on a five-story shear building to assess the performance of DWT based detection approach. The made observations indicate that the DWT based detection index proposed in this study can accurately identify the damage time instant and damage location due to a sudden stiffness reduction in terms of the occurrence time and spatial distribution of damage index spikes. The proposed damage index is linearly proportional to damage severity, but the slope of the linear function depends on external excitation and damage time instant. A possible way of determining damage severity has been suggested using the calibrated structural model and the measured excitation. The proposed damage index can identify the damage events from the contaminated acceleration responses if the noise frequency range is limited. If the noise frequency range is wide enough, the reliability of damage detection using the proposed approach remarkably deteriorates with the increase of noise intensity.

## Figures and Tables

**Table 1:** Natural frequency before and after sudden damage.

Damage extent	Frequencies (Hz)				
	*f*_1_	*f*_2_	*f*_3_	*f*_4_	*f*_5_
0%	2.513	7.335	11.563	14.854	16.941
1%	2.508 (−0.18%)	7.324 (−0.15%)	11.55 (−0.11%)	14.85 (−0.05%)	16.94 (−0.01%)
2%	2.504 (−0.36%)	7.313 (−0.30%)	11.54 (−0.21%)	14.84 (−0.11%)	16.94 (−0.03%)
5%	2.490 (−0.94%)	7.278 (−0.78%)	11.50 (−0.53%)	14.81 (−0.26%)	16.93 (−0.07%)
10%	2.464 (−1.97%)	7.218 (−1.62%)	11.44 (−1.06%)	14.78 (−0.52%)	16.92 (−0.14%)
20%	2.407 (−4.41%)	7.088 (−3.48%)	11.32 (−2.18%)	14.71 (−1.01%)	16.90 (−0.26%)
40%	2.253 (−11.6%)	6.781 (−8.16%)	11.07 (−4.50%)	14.57 (−1.92%)	16.86 (−0.46%)

Note: values in brackets are the percentage of change in natural frequency.

**Table 2:** Variations of damage index with damage severity.

Damage severity	1%	2%	5%	10%	20%	40%
DI (seismic excitation)	2.625	5.223	13.017	26.004	51.960	103.814
DI (sinusoidal excitation)	5.172	10.344	25.857	51.704	103.369	206.579
DI (impulse excitation)	1.256	2.519	6.309	12.623	25.244	50.457
